# A meta-analysis comparing cognitive function between individuals at clinical high-risk for psychosis and individuals at family high-risk for psychosis

**DOI:** 10.1186/s12888-025-07717-z

**Published:** 2025-12-24

**Authors:** Ronghong Gao, Mengmeng Fan, Anpei wei, Ting Liu, Lili Guo, Xiaoyan He, Zhaoguo Liu

**Affiliations:** 1 Affiliated Mental Health Center of Jiangnan University, Wuxi Central Rehabilitation Hospital, Wuxi City, 214151 China; 2 School of Humanities and Management science, Wannan Medical College, Wuhu City, 241002 China; 3 Wuxi Huishan District People’s Hospital, Wuxi City, 214187 China

**Keywords:** Clinical high risk (CHR), Familial high risk (FHR), Cognition, Processing speed, Verbal learning, MATRICS, Meta-analysis

## Abstract

**Background:**

Cognitive impairment is detectable before psychosis onset, yet no quantitative synthesis has directly contrasted neurocognition between clinical high risk (CHR) and familial high risk (FHR). We aimed to clarify domain-level differences to inform assessment and early intervention.

**Methods:**

Following PRISMA/MOOSE, we searched PubMed, Embase, and PsycINFO (1996–May 2025) for peer-reviewed studies reporting standardized neurocognitive outcomes in both CHR and FHR groups. Outcomes were organized by MATRICS domains. Effect sizes were Hedges’ g (coded CHR − FHR; negative = poorer CHR) synthesized with random-effects. When multiple outcomes occurred within a study/domain, a within-study fixed-effects composite (*r* = 0.50) was used. Heterogeneity (Q, I²), small-study effects (Egger’s test), and prespecified meta-regression (when k ≥ 10) were performed.

**Results:**

Fourteen studies were included (CHR *n* = 1,160; FHR *n* = 813). Processing speed: CHR underperformed FHR (pooled g = − 0.290, 95% CI: −0.521 to − 0.059, *p* = 0.014;); task-level analyses showed significant effects for TMT-A and Stroop baselines. Attention, working memory, executive function, and visual learning showed no robust between-group differences at the domain level (all *p* > 0.10; moderate–high heterogeneity). Verbal learning: subgroups indicated CHR deficits in list-learning—immediate (g = − 0.558, 95% CI − 1.099 to − 0.017, *p* = 0.043) and list-learning—delayed (g = − 0.296, 95% CI − 0.526 to − 0.067, *p* = 0.011). Wechsler Memory Scale (WMS) Logical Memory (immediate/delayed) was under-represented (*k* < 3). Meta-regression (processing speed): region moderated effects (Non-Asian vs. Asian: β = 0.486, 95% CI 0.051–0.921, *p* = 0.0287; R²_analog ≈ 24%); age difference, publication year, and study quality were not significant. Egger’s tests did not indicate small-study effects (all *p* > 0.05).

**Conclusions:**

Compared with FHR, CHR shows reliable impairments in processing speed and verbal list-learning, while other domains do not differ robustly. Findings refine the cognitive phenotype of CHR beyond familial liability and highlight processing speed and list-learning as pragmatic markers for risk stratification and monitoring. Clinical trial number: not applicable.

**Supplementary Information:**

The online version contains supplementary material available at 10.1186/s12888-025-07717-z.

## Introduction

Schizophrenia is a chronic psychiatric disorder that typically emerges in late adolescence or early adulthood; the median lifetime prevalence is ~ 0.4% (4 per 1,000) and the lifetime morbid risk is ~ 0.7% (7.2 per 1,000) in population-based studies [1]. Cognitive dysfunction is widely recognized as a core feature of psychotic disorders, with evidence showing it is present even in antipsychotic-naive first-episode patients and varies substantially between individuals, in ways that predict functional outcomes [2]. Extensive meta-analytic data show that the duration of illness is associated with long-term cognitive decline, particularly in schizophrenia spectrum disorders [3]. Impaired cognition at disease onset has also been linked to poorer response to antipsychotic treatments [4].

A substantial body of research demonstrates that cognitive abnormalities precede the onset of psychosis and evolve dynamically across development, supporting neurodevelopmental vulnerability models [5]. Population-based birth-cohort studies demonstrate that individuals who later develop schizophrenia exhibit early-emerging deficits—particularly in verbal and crystallized cognitive abilities—that arise in childhood and remain relatively stable thereafter, alongside progressively increasing developmental lag in later-maturing domains such as processing speed, working memory, visuospatial reasoning, and executive functions across adolescence and early adulthood. Importantly, these findings indicate slower developmental growth relative to age-matched norms rather than frank cognitive deterioration [6]. These cognitive discrepancies frequently widen as individuals approach the prodromal phase. For example, longitudinal birth-cohort studies have shown that individuals who develop schizophrenia exhibit pronounced decline in processing speed and other nonverbal cognitive functions across development, while verbal abilities and delayed memory show early-emerging deficits that remain relatively stable over time [7], while Reichenberg et al. demonstrated early and stable premorbid deficits in verbal and knowledge-based functions alongside developmental lag in processing speed, working memory, and visuospatial abilities from ages 7 to 13 years in individuals who later developed schizophrenia [8]. Complementary commentary has cautioned that apparent declines in standardized IQ scores may partly reflect slower cognitive growth relative to age-matched norms, rather than frank loss of cognitive ability, underscoring the importance of distinguishing developmental lag from true cognitive deterioration when interpreting longitudinal trajectories [9].

Within this developmental context, two major paradigms have been used to examine cognitive functioning before psychosis onset: the familial high-risk (FHR) and clinical high-risk (CHR) approaches. These paradigms capture different points along the psychosis-risk continuum and can be interpreted in light of the clinical staging model. FHR individuals, defined by having a first-degree relative with a psychotic disorder, represent a population characterized primarily by genetic or familial vulnerability in the absence of manifest psychotic symptoms. Cognitive alterations observed in FHR samples are therefore often conceptualized as trait-like and developmentally stable, reflecting early neurodevelopmental deviations rather than active disease processes. In contrast, CHR individuals are identified based on attenuated psychotic symptoms, brief limited psychotic episodes, and/or recent functional decline, and are commonly viewed as occupying an early prodromal stage marked by dynamic pathophysiological changes and heightened short-term risk of transition to psychosis [10, 11].

Consistent with this framework, numerous studies show that CHR individuals perform worse than healthy controls, with cognitive profiles that are intermediate between those of first-episode schizophrenia and controls [10, 12–15]. Within CHR cohorts, poorer cognition—especially in processing speed, verbal learning and memory, and global IQ—has been associated with higher risk of transition to psychosis [12, 13], and recent longitudinal work suggests that within-individual change in cognition may be a more sensitive marker of risk progression than baseline performance alone [16]. At the same time, meta-analytic and family studies in FHR samples indicate smaller but reliable impairments and a strong genetic correlation between cognitive ability and schizophrenia liability, supporting cognition as an endophenotype for psychosis risk [14, 17, 18].

Recent neuroimaging evidence has also begun to examine differences across risk groups. For example, a recent meta-analysis of functional magnetic resonance imaging(fMRI) studies reported distinct neural activation patterns related to social cognition across CHR, FHR, and first-episode psychosis groups, suggesting that these populations may follow different neural pathways toward psychosis [19]. Another meta-analytic review of structural and functional brain alterations supports decreased grey matter volume and hypoactivation patterns in CHR vs FHR vs healthy controls(HC) [20]. While such imaging-based evidence provides valuable insights into the neural correlates of risk states, it does not directly address behavioral manifestations of cognition. To date, no meta-analysis has synthesized behavioral neurocognitive performance specifically comparing CHR vs FHR groups (though CHR vs HC and FHR vs HC have been compared) [14].”

Therefore, the present study aims to fill this gap by synthesizing evidence from studies published between 1996 and May 2025. Through a meta-analytic approach, we seek to clarify differences in cognitive functioning between CHR and FHR populations and provide evidence-based implications for clinical assessment and early intervention strategies.

## Methods

### Study registration and reporting

This meta-analysis was registered in PROSPERO (registration number: CRD42024557162) and conducted in accordance with the Preferred Reporting Items for Systematic Reviews and Meta-Analyses (PRISMA) [21] and the Meta-analysis of Observational Studies in Epidemiology (MOOSE) guidelines [22] (Supplementary Tables 1–2).

### Search strategy and study selection

We systematically searched PubMed, Embase, and PsycINFO for eligible studies published between January 1, 1996, and May 31, 2025, as the construct of CHR was formally defined after 1996. Searches were restricted to publications with English abstracts. The Boolean strategy combined three components:

- **Population**: (“at risk mental state” OR ARMS OR “ultra-high risk” OR UHR OR “clinical high risk” OR CHR OR “psychosis risk” OR prodrome OR psychosis OR “basic symptoms”)
- **Comparison group**: (“familial high risk” OR FHR OR “first-degree relatives” OR FDR OR “genetically high risk” OR GHR)
- **Outcomes**: (cognition OR cognitive OR neurocognitive OR neuropsychological OR neurocognition).

Reference lists of relevant reviews and included articles were hand-searched for additional records. Two psychiatrists independently screened titles/abstracts and then full texts. Disagreements were resolved by discussion; a senior reviewer arbitrated when necessary.

### Inclusion and exclusion criteria

**Inclusion criteria:**

- Original peer-reviewed articles in English;
- Quantitative neurocognitive test results;
- Clearly defined FHR as unaffected first-degree relatives of patients with schizophrenia;
- CHR defined using validated instruments;
- Cognitive data reported for both CHR and FHR groups.

**Exclusion criteria:**

- Non-standard or ambiguous CHR definitions;
- Overlapping samples on the same cognitive measures;
- Studies reporting only FHR or only CHR;
- Abstracts, pilot datasets, conference proceedings, or non-English publications.

### Data extraction

Two reviewers independently extracted data using a structured template. Extracted variables included first author, publication year, country, and group-specific characteristics (numbers of CHR and FHR participants, mean age, sex distribution), as well as details of cognitive assessments. Discrepancies were resolved by consensus or by a senior reviewer if needed.

### Quality assessment

Study quality was evaluated using the Newcastle–Ottawa Scale (NOS), assessing selection, comparability, and outcome domains (score range 0–9). Ratings are shown in Supplementary Table 3.

### Outcome measures

Neurocognitive outcomes were grouped into domains according to the MATRICS framework and previous CHR meta-analyses [10, 15], covering: processing speed, attention, working memory, verbal learning and memory, visual learning, and executive function. Tasks reported by ≥ 3 independent studies were meta-analyzed separately; tasks reported by fewer studies contributed only to domain-level pooled estimates. The task-to-domain mapping is detailed in Supplementary Table 4.

### Statistical analysis

Effect sizes were calculated as standardized mean differences (SMD; Hedges’ g) with 95% confidence intervals (CIs). For each individual study, Hedges’ g was computed; when pooling across studies, random-effects models (DerSimonian–Laird) [23] were used to obtain the overall SMD. Effect sizes were coded as CHR – FHR, such that negative values indicate poorer performance in CHR relative to FHR.

For score/accuracy outcomes, higher values indicated better performance. For time/error outcomes, values were reverse-coded so that higher values consistently reflected better performance across all measures. When multiple outcomes from the same cognitive domain were reported within a study, a single within-study effect was created using a fixed-effects composite with inverse-variance weights, assuming an inter-outcome correlation of *r* = 0.50. This ensured one effect per study for each meta-analysis. Sensitivity analyses varying r were not undertaken. Heterogeneity was assessed using Cochran’s Q test (*p* < 0.10 denoting significance) and the I² statistic. Sensitivity analyses were conducted via leave-one-out procedures. Publication bias was evaluated using funnel plots and Egger’s regression when ≥ 10 studies were available for a given analysis.

### Meta-regression analyses

Exploratory meta-regression was planned a priori to investigate potential sources of heterogeneity when sufficient effect sizes (≥ 10) were available within a domain. Prespecified moderators included: (a) mean age difference between CHR and FHR groups, (b) publication year(centred), (c) geographical region, and (d) study quality (NOS score). Random-effects meta-regression models were used, with significance set at *p* < 0.05.

All analyses were conducted using Comprehensive Meta-Analysis (CMA v3.0; Biostat, USA) and IBM SPSS Statistics v20.0 (IBM Corp., Armonk, NY, USA).

## Results

### Study selection

**Fig. 1:**
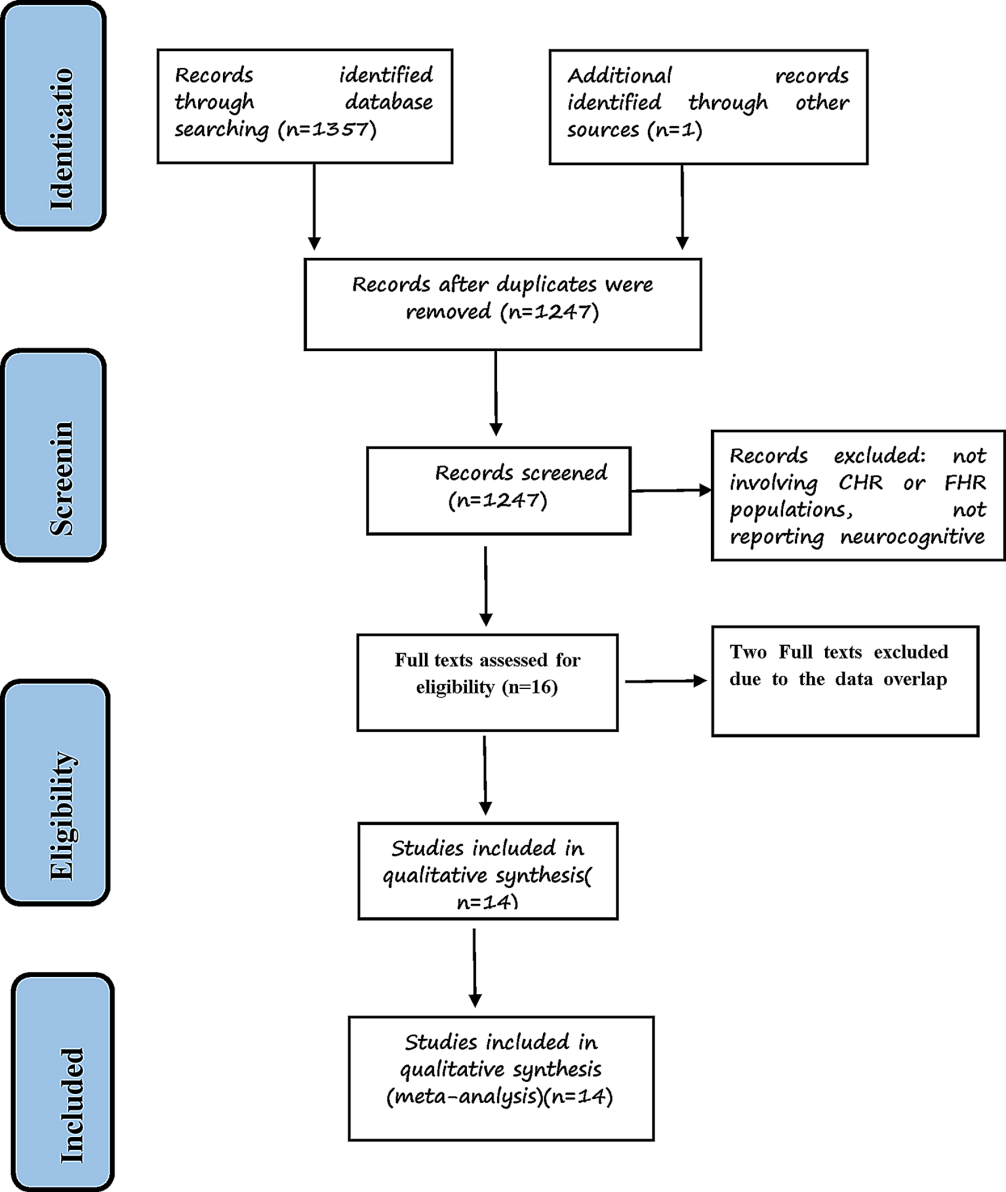
PRISMA flow diagram of study selection for the meta-analysis of cognitive performance in clinical high risk (CHR) versus familial high risk (FHR)

A total of 1,358 records were identified through database searches (PubMed = 1,100; Embase = 191; PsycINFO = 56) and other resources. After removing 111 duplicates, 1,247 records remained for title and abstract screening. Of these, 1,231 were excluded because they did not meet the eligibility criteria (e.g., not involving CHR or FHR populations, not reporting neurocognitive outcomes). The full texts of the remaining 16 articles were reviewed in detail. Among them, 2 studies was excluded due to overlapping datasets, resulting in 14 studies included in the final meta-analysis (Fig. 1).

### Study characteristics

The included studies were published between 2007 and 2025 across multiple countries (e.g., China, USA, Australia, Europe). Sample sizes ranged from 20 to 338, with a total of *N* = 1,160 CHR individuals and *N* = 813 FHR individuals. The mean age was 21.1 years (SD 4.6) in the CHR group and 29.1 years (SD 9.0) in the FHR group. The proportion of males ranged from 25% to 64% across studies. CHR diagnosis was mostly based on CAARMS or SIPS. Cognitive outcomes covered the MATRICS domains, assessed by standardized tests such as the Trail Making Test (TMT-A/B), Digit Symbol Coding, Continuous Performance Test (CPT-IP), Digit Span, California Verbal Learning Test (CVLT), Rey Auditory Verbal Learning Test (RAVLT) Rey–Osterrieth Complex Figure Test, and WCST. A summary of study characteristics is presented in Table 1.

**Table 1:** Neurocognitive domains, representative tasks, and number of studies included in the meta-analysis

Study(year)	Country	Sample size, *N*(male)		Age, M(SD)		NOS	Tasks analysed
		CHR	FHR	CHR	FHR		
Myles-Worsley et al. (2007)	USA	113 (51)	98 (46)	17.2 (1.2)	16.9 (2.1)	5	WISC-III, WMS, CPT-IP, Lincoln–Oseretsky
Seidman et al. (2010)	USA-Canada	167 (107)	49 (23)	18.2 (4.9)	18.7 (4.1)	6	Vocabulary, Block Design, CPI-IP, TMT- B, WCST, COWAT, HVLT, CVLT, RAVLT
Mukkala et al. (2011)	Finland	20 (5)	62 (28)	22.7 (0.9)	22.8 (0.7)	6	WAIS-III WMS-R CVLT, CANTAB, Semantic fluency, Sternberg working memory
Üçok 2013 et al. (2013)	Tukey	52 (36)	29 (13)	21.20 (5.16)	23.79 (6.88)	5	Rey verbal learning test, TMT, WCST, SCWT, CPT, Digit Span
Hou et al. (2016)	China	40 (16)	40 (21)	29.1 (7.0)	25.3 (5.2)	7	DST, SCWT, TMT-A, HVLT-R
Kim et al. (2017)	Korea	32 (22)	32 (16)	20.6 (5.9)	24.8 (5.2)	7	TMT, WCST, CVLT, Digit span, Verbal fluency,
Chu et al. (2019)	Hong Kong	71 (43)	50 (21)	20.8 (6.5)	25.4 (6.3)	6	WAIS-R, WMS-R
Togay et al. (2019)	Turkey	29 (22)	24 (11)	20.21 (4.49)	27.62 (5.24)	6	WCST, SCWT, TMT
Liu et al.(2019)	China	73 (41)	44 (24)	23.3 (4.5)	25.6 (4.7)	7	Chinese MATRICS
DongFang et al. (2023)	China	42 (26)	26 (15)	23.8 (4.8)	26.7 (4.8)	7	Chinese MATRICS
Zadeh et al. (2023)	Iran	40 (12)	24 (10)	27.48 (5.34)	26.59 (4.85)	7	CANTAB, COWAT
Ouyang et al. (2024)	China	89 (49)	81 (41)	19.54 (4.35)	21.2 (5.56)	7	Chinese MATRICS
Delphine et al. (2024)	France	338 (217)	163 (85)	20.8 (3.41)	52.7 (17.3)	7	WAIS, Minicog
He et al. (2025)	China	54 (27)	80 (42)	28.24 (7.46)	27.21 (7.28)	7	TMT-A, DST, SWCT, BVMT-R, HVLT-R

Abbreviations: CANTAB = Cambridge Neuropsychological Test Automated Battery; CHR = Clinical High Risk (for psychosis); COWAT = Controlled Oral Word Association Test; CPT = Continuous Performance Test; CPT-IP = Continuous Performance Test – Identical Pairs; CVLT = California Verbal Learning Test; DSST (DST) = Digit Symbol Substitution Test (WAIS subtest); Digit Span (WAIS) = Wechsler Adult Intelligence Scale – Digit Span; FHR = Familial High Risk; HVLT / HVLT-R = Hopkins Verbal Learning Test / Revised, Lincoln–Oseretsky = Lincoln–Oseretsky Motor Development Scale; MATRICS / MCCB = MATRICS Consensus Cognitive Battery; NOS = Newcastle–Ottawa Scale; RAVLT = Rey Auditory Verbal Learning Test; SCWT (Stroop) = Stroop Color and Word Test; TMT / TMT-A / TMT-B = Trail Making Test / Part A / Part B; WAIS = Wechsler Adult Intelligence Scale; WAIS-III / WAIS-IV / WAIS-R = Third / Fourth Edition / Revised; WCST = Wisconsin Card Sorting Test; WISC-III = Wechsler Intelligence Scale for Children – Third Edition; WMS / WMS-R = Wechsler Memory Scale / Revised; Benton–Hamsher tests = e.g., Judgment of Line Orientation, Facial Recognition, Visual Retention

**Table 2:** Meta-analysis results by domain

Test or outcome measure	CHR No	FHR No	Studies	SMD/ Hedges’ g (95% CI)	*p*	Heterogeneity		Publication Bias
			No			Q	I^2^	Eggers
**Processing speed**								
TMT-A	634	450	7	-0.386(-0.663~-0.110)	**0.006**	23.95	74.95	0.14
Digit symbol coding	332	219	4	-0.572(-1.224~0.080)	0.086	36.71	91.83	0.21
SCWT (word or color)	264	255	5	-0.475(-0.913~-0.036)	**0.034**	28.5	85.97	0.99
Processing speed pooled variability estimate	953	730	12	-0.290(-0.521~-0.059)	**0.014**	66.64	83.49	0.36
**Attention**								
CPI	475	338	5	-0.329(-0.778~0.120)	0.151	32.32	87.59	0.68
Attention pooled variability estimate	681	520	9	-0.143(-0.457~0.17)	0.371	59.55	86.56	0.78
**Working memory**								
digit span	217	252	4	0.173(-0.095~0.440)	0.206	5.39	44.38	0.53
Working memory pooled variability estimate	443	376	8	-0.131(-0.374~0.111)	0.288	22.51	68.9	0.3
**Executive function**								
TMT-B	707	379	6	-0.294(-0.658~0.070)	0.113	32.72	84.72	0.64
WCST	280	135	4	-0.455(-1.509~0.598)	0.397	75.44	96.02	0.47
Executive function pooled variability estimate	1160	831	14	-0.170(-0.377~0.037)	0.108	71.11	81.72	0.07
**Verbal learning**								
HVLT	183	201	3	-0.994(-1.882~-0.106)	**0.028**	35.12	94.31	0.07
Logical Memory	204	210	3	0.299 (0.119~0.479)	**0.001**	1.6·	0	0.45
Verbal learning pooled variability estimate	753	592	11	/	**/**	/	/	/
**Visual learning**								
Visual learning pooled variability estimate	520	347	6	0.4998(-0.536~1.531)	0.345	248.91	97.99	0.42

Bold values: P£0.05. Abbreviations: CHR = Clinical High Risk; CPT = Continuous Performance Test; FHR = Familial High Risk; HVLT = Hopkins Verbal Learning Test; SCWT = Stroop Color and Word Test (word & color naming); SMD (Hedges’ g) = standardized mean difference WCST = Wisconsin Card Sorting Test; TMT = Trail Making Test

Note: Verbal learning/memory was not pooled due to opposing directions between test families and high heterogeneity; list-learning subgroups (immediate/delayed) are reported in Supplementary Table 5, whereas WMS Logical Memory had fewer than three studies and was not meta-analyzed

### Quality assessment

Using the Newcastle–Ottawa Scale (NOS), all studies were rated as moderate-to-high quality, with scores ranging from 5 to 7 (Table 1).

### Main effects

#### Processing speed

**Fig. 2:**
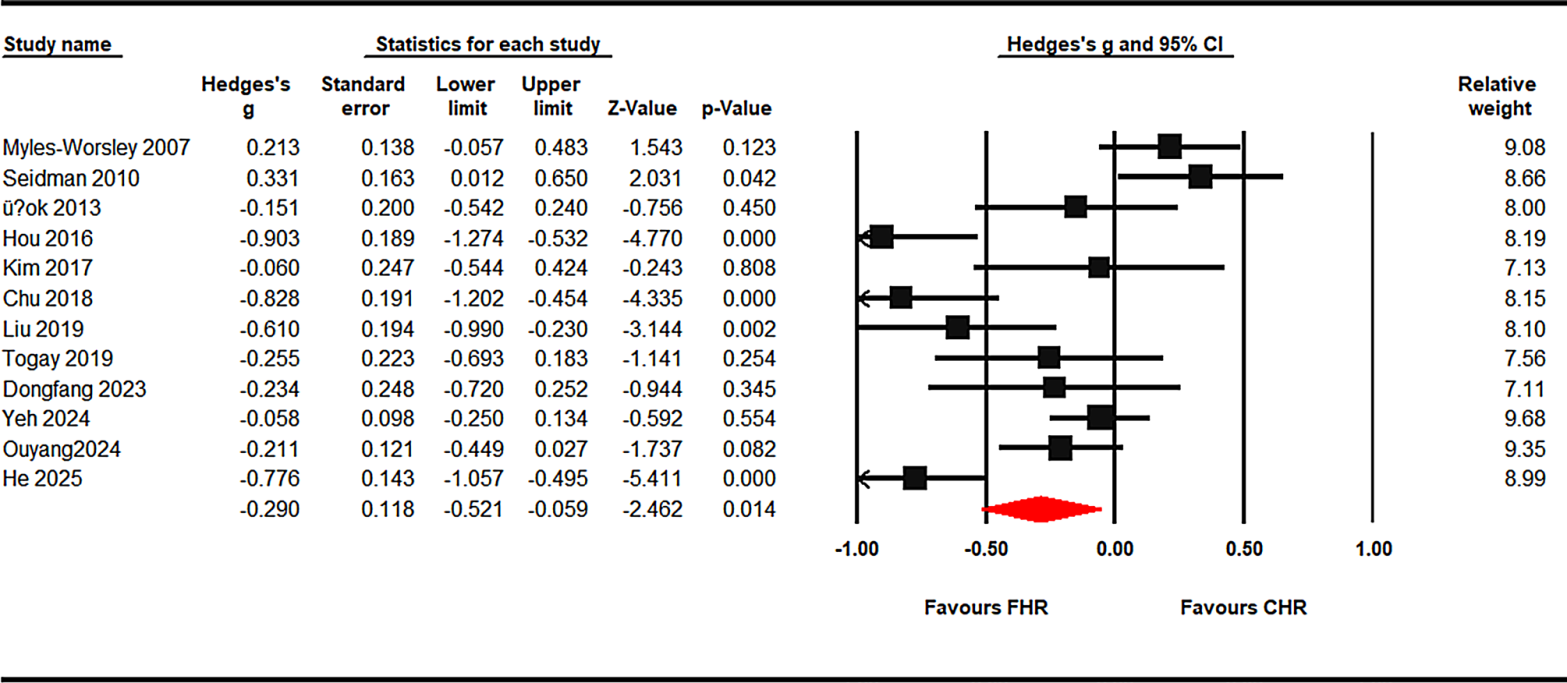
Processing speed: CHR vs. FHR. Random-effects meta-analysis of Hedges’ g (CHR − FHR); negative values favor FHR. The diamond shows the pooled effect with 95% CI. Prediction interval (95%): [-1.147,0.566]

Across k = 12 studies, CHR showed a significant deficit in processing speed relative to FHR (pooled g = − 0.290, 95% CI − 0.521 to − 0.059, *p* = 0.014; I² ≈ 83%). At the task level, TMT-A was significant (g = − 0.389, 95% CI − 0.668 to − 0.111, *p* = 0.006), Stroop baseline (word/color) was also significant (g = − 0.475, 95% CI − 0.913 to − 0.036, *p* = 0.034), whereas Digit Symbol/Coding showed a non-significant trend (g = − 0.572, 95% CI − 1.224 to 0.080, *p* = 0.086) (Fig. 2 and supplement Figs. 1 and 2).

#### Attention

Nine studies contributed attention outcomes (e.g., CPT). The pooled effect was not significant (g = − 0.143, 95% CI − 0.457 to 0.170, *p* = 0.371; I² ≈ 87%) (see supplement Fig. 3).

**Fig. 3:**
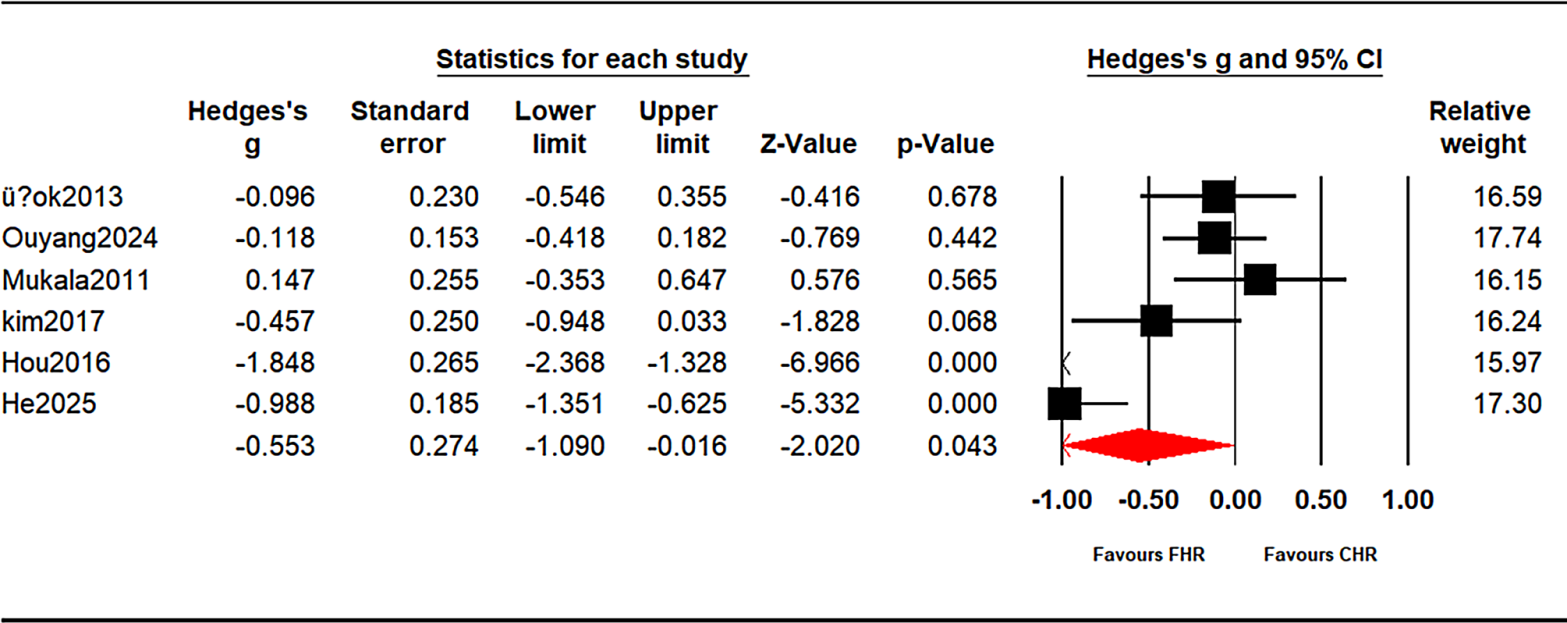
Verbal learning(listen-learning immediate): CHR vs. FHR. Random-effects meta-analysis of Hedges’ g (CHR − FHR); negative values favor FHR. The diamond shows the pooled effect with 95% CI. Prediction interval (95%): [-2.466, 1.359]

#### Working memory

Eight studies assessed working memory (e.g., Digit Span, Spatial Span). The pooled effect was non-significant (g = − 0.131, 95% CI − 0.374 to 0.111, *p* = 0.288; I² ≈ 69%) (see supplement Fig. 4).

**Fig. 4:**
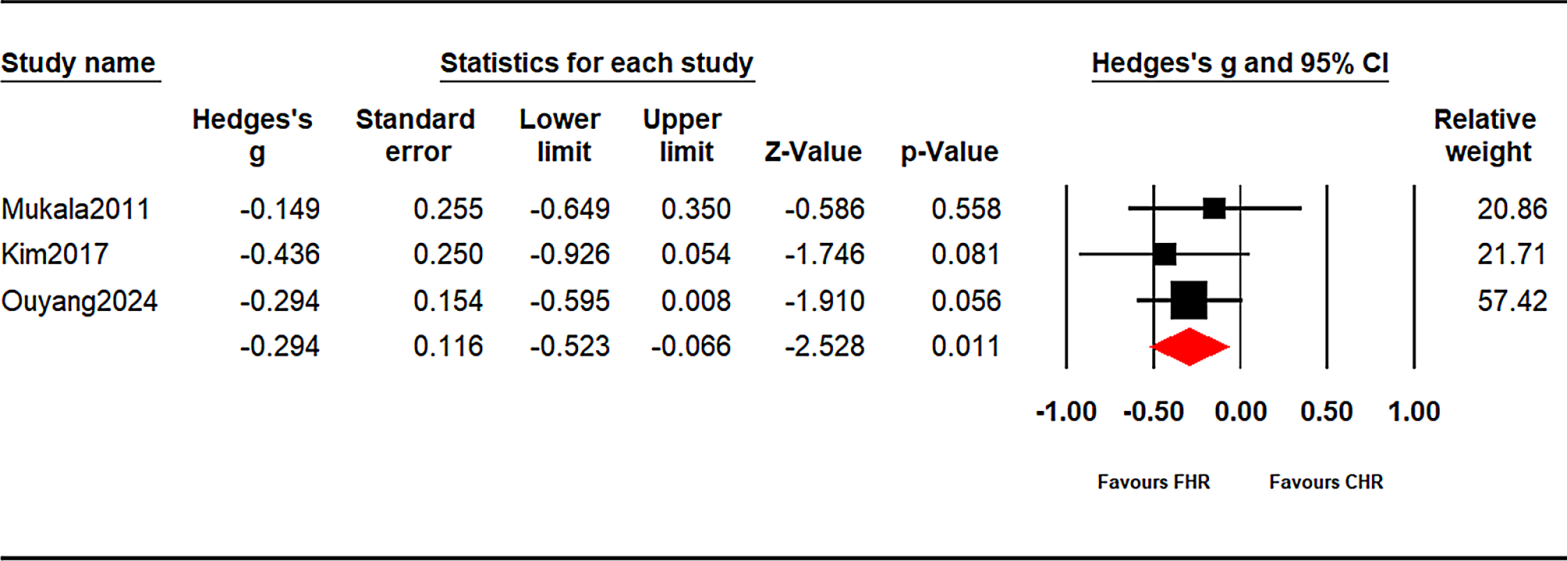
Verbal learning(listen-learning delayed): CHR vs. FHR. Random-effects meta-analysis of Hedges’ g (CHR − FHR); negative values favor FHR. The diamond shows the pooled effect with 95% CI. Prediction interval (95%): [-, UL]

#### Executive function

Across k = 14 studies (e.g., TMT-B, WCST), the pooled effect was non-significant (g = − 0.170, 95% CI − 0.377 to 0.037, *p* = 0.108; I² ≈ 82%) (see supplement Fig. 5).

**Fig. 5:**
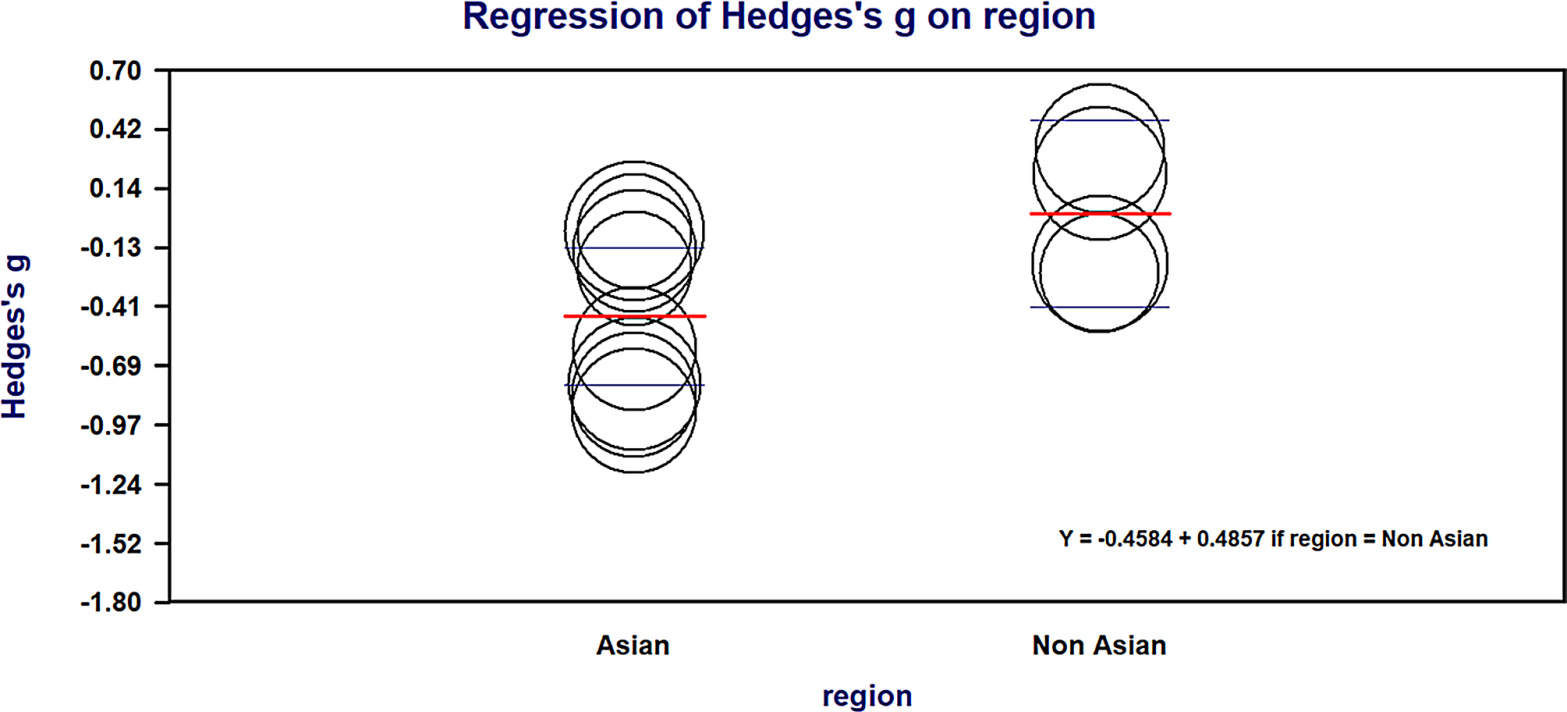
Meta-regression for processing speed by region. Bubble size is proportional to inverse-variance weight. Fitted means (horizontal bars) are shown for Asian (0) and Non-Asian (1). Random-effects meta-regression: β = 0.486, 95% CI 0.051–0.921, *p* = 0.0287; R²_analog = 24%; k = 24. Effects are Hedges’ g (CHR − FHR); negative values indicate poorer CHR performance (favours FHR)

#### Verbal learning (prespecified subgroups)

Because test families pointed in opposite directions and contributed to high heterogeneity, verbal outcomes were analyzed a priori by subgroup (see Supplementary Tables 5 and Figs. 3 and 4):

- List-learning — Immediate (HVLT/CVLT/RAVLT immediate/total; k = 6): g = − 0.558, 95% CI − 1.099 to − 0.017, *p* = 0.043.
- List-learning — Delayed (HVLT/CVLT/RAVLT delayed; k = 3): g = − 0.296, 95% CI − 0.526 to − 0.067, *p* = 0.011.
- Wechsler Memory Scale (WMS) Logical Memory (immediate/delayed): k < 3, therefore not meta-analyzed;

#### Visual learning

Six studies examined visual learning/memory; results were inconsistent and the pooled effect was non-significant (g = + 0.500, 95% CI − 0.536 to 1.531, *p* = 0.345; I² ≈ 98%) (see supplement Fig. 6).

#### Heterogeneity and sensitivity analyses

Substantial between-study heterogeneity was observed in several domains (processing speed I² ≈ 83%, attention ≈ 87%, working memory ≈ 69%, executive function ≈ 90%, visual learning/memory ≈ 98%). Leave-one-out analyses did not materially change the pooled effects or their significance in any domain. For verbal learning/memory, the prespecified subgroups reduced conceptual heterogeneity (list-learning immediate and delayed were consistent in direction), whereas WMS Logical Memory remained under-represented (k < 3) and was not pooled (See Table 2).

#### Publication bias

Small-study effects were examined where k ≥ 10. Funnel plots for processing speed and executive function were visually symmetrical, and Egger’s regression did not indicate significant small-study effects (two-sided *p* > 0.05 for both). No formal tests were conducted for other domains (k < 10) (See Table 2).

#### Meta-regression (processing speed)

Exploratory univariate random-effects meta-regressions were performed for processing speed. Region was the only significant moderator: studies conducted in non-Asian settings showed a higher (less negative) effect relative to Asian studies (β = 0.486, SE = 0.222, 95% CI 0.051 to 0.921, z = 2.19, *p* = 0.0287), explaining about 24% of the between-study variance (R²_analog ≈ 24%)(See Fig. 5). Age difference between CHR and FHR (β = −0.010, *p* = 0.473), publication year (centred) (β = −0.032, *p* = 0.108), and study quality (NOS) (β = −0.210, *p* = 0.155) were not significant. These findings suggest that geographical setting partly accounts for variability in processing-speed differences, whereas age imbalance, recency of publication, and study quality do not (See Table 3).

**Table 3:** Univariate random-effects meta-regressions for processing speed (CHR vs. FHR)

Moderator	Beta	SE	95%	95%	Z-value	*P*-value	*R*² analog (%)
			CI lower	CI upper			
Age difference, years (CHR-FHR)	-0.01	0.014	-0.037	0.017	-0.72	0.473	0
Publication year(centered)	-0.032	0.02	-0.072	0.007	-1.61	0.108	8
Region (No Asian=1, Asian=0)	0.486	0.222	0.051	0.921	2.19	**0.0287**	24
Study quality (NOS, per 1-point)	-0.21	0.147	-0.498	0.079	-1.42	0.155	9

Bold values: P£0.05.Abbreviations: CHR = Clinical High Risk; FHR = Familial High Risk; NOS = Newcastle-Ottawa Scale

## Discussion

### Principal findings

Across 14 studies directly comparing CHR and FHR individuals, we identified robust deficits in processing speed and verbal list-learning in CHR relative to FHR, whereas attention, working memory, executive function, and visual learning/memory showed no consistent group differences at the domain level. Within verbal learning/memory, list-learning (HVLT/CVLT/RAVLT) was impaired for both immediate acquisition and delayed recall, while WMS Logical Memory showed opposite trends but was under-represented (k < 3), precluding domain-level pooling. Meta-regression for processing speed indicated that region moderated effects—CHR–FHR differences were smaller in non-Asian than in Asian samples—whereas age difference, publication year, and study quality (NOS) were not significant moderators.

### Interpretation and context

A key contribution of the present study is that its pattern of findings accords closely with longitudinal evidence on neurocognitive development across the schizophrenia spectrum. Converging population-based cohort studies indicate that cognitive dysfunction associated with later psychosis is not abrupt, but unfolds gradually across development through partially dissociable processes. Specifically, early-emerging and relatively stable cognitive deviations—particularly in verbal abilities, reasoning, and other measures of crystallized knowledge—are detectable from childhood, whereas domains such as processing speed, working memory, and executive functioning show slower developmental gains and widening gaps relative to peers across adolescence and early adulthood [5, 7, 8].

Importantly, longitudinal investigations further demonstrate that individuals who later develop psychosis exhibit amplification of these differences as they approach the prodromal period, with the most consistent proximity-related changes observed in processing speed, verbal learning, and working memory [6]. When placed within this developmental context, the present meta-analytic findings—showing greater impairment in CHR than in FHR specifically in processing speed and verbal list-learning—are consistent with domains that have repeatedly been identified as particularly sensitive to the period approaching psychosis onset.

When interpreted within a developmental and clinical staging framework, the observed CHR–FHR differences are consistent with models proposing that these two groups reflect different positions along the psychosis-risk continuum. FHR samples primarily index inherited or familial vulnerability in the absence of manifest psychotic symptoms, and cognitive alterations observed in this group are therefore commonly conceptualized as trait-like and relatively stable across development. In contrast, CHR samples capture individuals experiencing attenuated psychotic symptoms and/or functional decline, a clinical state widely considered to reflect an active prodromal phase associated with heightened short-term risk of transition to psychosis.

From this perspective, the additional impairments observed in CHR—particularly in processing speed and verbal list-learning—may reflect the superimposition of emerging state-related cognitive inefficiency on top of shared familial or developmental vulnerability. Thus, beyond shared familial vulnerability, our findings indicate that CHR samples exhibit additional domain-specific impairments relative to FHR, consistent with closer proximity to illness onset.

### Clinical and research implications

Processing speed and verbal list-learning emerged as priority cognitive domains differentiating CHR from FHR, offering clinically relevant insights when interpreted within a developmental framework and related clinical staging models. Rather than serving as diagnostic markers, these domains may help characterize relative proximity along the psychosis-risk continuum. In settings where individuals with familial risk are monitored, relative slowing in processing speed or reduced efficiency in verbal list-learning may may help characterise individuals exhibiting cognitive profiles more similar to those observed in CHR samples and inform decisions about closer clinical monitoring. Brief, well-established measures such as the Trail Making Test–A or Digit Symbol/Coding tasks are widely available and have been shown to be sensitive to cognitive changes associated with the prodromal phase of psychosis [24].

For CHR samples, cognitive remediation and compensatory interventions targeting processing efficiency and learning strategies may be particularly relevant. P rior meta-analyses have demonstrated that cognitive remediation interventions targeting processing speed and learning are associated with measurable cognitive and functional benefits in schizophrenia, including early-stage samples [25, 26], suggesting potential relevance during the prodromal phase.

Finally, the observed regional moderation effects underscore the importance of considering contextual factors—such as educational background, test familiarity, and cultural–linguistic influences—when interpreting cognitive differences across studies or designing multisite and cross-regional research [27, 28].

### Heterogeneity and robustness

Between-study heterogeneity was substantial (I² often > 70%), likely reflecting variability in tasks, sampling, and procedures. Our strategy of one effect per study and construct-based subgrouping reduced noise and yielded stable inferences. Leave-one-out analyses confirmed robustness, and Egger’s tests did not suggest publication bias (all *p* > 0.05).

### Strengths and limitations

This review provides the first direct quantitative synthesis of CHR versus FHR cognition, with pre-specified handling of verbal constructs and meta-regression of moderators. However, high heterogeneity, small subgroup sizes (especially Logical Memory), and unmeasured clinical moderators limit interpretation. Between-study heterogeneity was substantial (I² > 70% in several domains), reflecting task variability, sampling differences, and procedural inconsistencies. Our analytic strategy—one effect per study (within-study fixed-effects composites, *r* = 0.50) and construct-driven subgrouping for verbal learning—reduced conceptual noise and yielded more stable inferences. Leave-one-out analyses confirmed robustness, and Egger’s tests did not suggest publication bias (all *p* > 0.05), though power was limited with small k. Finally, the literature search was restricted to English-language publications, which may have resulted in language bias and the omission of relevant studies published in other languages.

### Future directions

Future research should harmonise test batterie, report comparable metrics (e.g., locally normed z-scores), and incorporate clinical covariates such as negative symptoms, functioning, and conversion outcomes to clarify mechanistic links. Trials of cognitive remediation targeting processing speed and list-learning may further inform early intervention strategies, with FHR individuals serving as a familial-liability comparator.

## Conclusion

In summary, CHR individuals show greater impairment than FHR in processing speed and verbal list-learning, while other domains reveal no consistent differences. These findings refine the cognitive phenotype of CHR beyond familial liability and highlight practical markers that may support clinical decision-making and risk stratification in early psychosis.

## Supplementary Information

Below is the link to the electronic supplementary material.

Supplementary Material 1

## Data Availability

The data are provided within the manuscript and supplementary information files.
