# Supplementary material for: A meta-analysis comparing cognitive function between individuals at clinical high-risk for psychosis and individuals at family high-risk for psychosis
Source: BMC Psychiatry. 2025 Dec 24;25:1205. doi: 10.1186/s12888-025-07717-z (PMC12754966; doi:10.1186/s12888-025-07717-z)
Supplement: Supplementary file 1 — Supplementary Material 1 [file 12888_2025_7717_MOESM1_ESM.docx]

# Supplementary Online Content

# Supplement Table 1. PRISMA Statement and Checklist

# Supplement Table 2. MOOSE Checklist

# Supplement Table 3. Risk of bias (quality) assessment using modified Newcastle-Ottawa Scale for cross-sectional and cohort studies

# Supplement Table 4. List of All Tests and Outcome Measures Used

# Supplement Table 5. Verbal learning subgroup meta-analyses

# Supplement figure1-6 Forest Plot

# Supplement Methods 7-9. Scatter plot

# Supplement Table 1. PRISMA statement and checklist

| **Section and Topic** | **Item #** | **Checklist item** | **Location**  **where item is reported** |
| --- | --- | --- | --- |
| **TITLE** | | |  |
| Title | 1 | Identify the report as a systematic review. | Cover page |
| **ABSTRACT** | | |  |
| Abstract | 2 | See the PRISMA 2020 for Abstracts checklist. | Abstract |
| **INTRODUCTION** | | |  |
| Rationale | 3 | Describe the rationale for the review in the context of existing knowledge. | Introduction |
| Objectives | 4 | Provide an explicit statement of the objective(s) or question(s) the review addresses. | Introduction |
| **METHODS** | | |  |
| Eligibility criteria | 5 | Specify the inclusion and exclusion criteria for the review and how studies were grouped for the syntheses. | Method |
| Information sources | 6 | Specify all databases, registers, websites, organisations, reference lists and other sources searched or consulted to identify studies. Specify the date when each source was last searched or consulted. | Method |
| Search strategy | 7 | Present the full search strategies for all databases, registers and websites, including any filters and limits used. | Method |
| Selection process | 8 | Specify the methods used to decide whether a study met the inclusion criteria of the review, including how many reviewers screened each record and each report retrieved, whether they worked independently, and if applicable, details of automation tools used in the process. | Method |
| Data collection process | 9 | Specify the methods used to collect data from reports, including how many reviewers collected data from each report, whether they worked independently, any processes for obtaining or confirming data from study investigators, and if applicable, details of automation tools used in the process. | Method |
| Data items | 10a | List and define all outcomes for which data were sought. Specify whether all results that were compatible with each outcome domain in each study were sought (e.g. for all measures, time points, analyses), and if not, the methods used to decide which results to collect. | Method |
|  | 10b | List and define all other variables for which data were sought (e.g. participant and intervention characteristics, funding sources). Describe any assumptions made about any missing or unclear information. | Method |
| Study risk of bias assessment | 11 | Specify the methods used to assess risk of bias in the included studies, including details of the tool(s) used, how many reviewers assessed each study and whether they worked independently, and if applicable, details of automation tools used in the process. | Method |
| Effect measures | 12 | Specify for each outcome the effect measure(s) (e.g. risk ratio, mean difference) used in the synthesis or presentation of results. | Method |
| Synthesis methods | 13a | Describe the processes used to decide which studies were eligible for each synthesis (e.g. tabulating the study intervention characteristics and comparing against the planned groups for each synthesis . | Method |
|  | 13b | Describe any methods required to prepare the data for presentation or synthesis, such as handling of missing summary statistics, or data conversions. | Method |

| **Section and Topic** | **Item #** | **Checklist item** | **Location**  **where item is reported** |
| --- | --- | --- | --- |
|  | 13c | Describe any methods used to tabulate or visually display results of individual studies and syntheses. | Method |
|  | 13d | Describe any methods used to synthesize results and provide a rationale for the choice(s). If meta-analysis was performed, describe the model(s), method(s) to identify the presence and extent of statistical heterogeneity, and software package(s) used. | Method |
|  | 13e | Describe any methods used to explore possible causes of heterogeneity among study results (e.g. subgroup analysis, meta- regression). | Method |
|  | 13f | Describe any sensitivity analyses conducted to assess robustness of the synthesized results. | Method |
| Reporting bias assessment | 14 | Describe any methods used to assess risk of bias due to missing results in a synthesis (arising from reporting biases). | Method |
| Certainty assessment | 15 | Describe any methods used to assess certainty (or confidence) in the body of evidence for an outcome. | Method |
| **RESULTS** | | |  |
| Study selection | 16a | Describe the results of the search and selection process, from the number of records identified in the search to the number of studies included in the review, ideally using a flow diagram. | Figure 1 |
|  | 16b | Cite studies that might appear to meet the inclusion criteria, but which were excluded, and explain why they were excluded. | Figure 1 |
| Study characteristics | 17 | Cite each included study and present its characteristics. | Table 1 |
| Risk of bias in studies | 18 | Present assessments of risk of bias for each included study. | Table 1 |
| Results of individual studies | 19 | For all outcomes, present, for each study: (a) summary statistics for each group (where appropriate) and (b) an effect estimate and its precision (e.g. confidence/credible interval), ideally using structured tables or plots. | Tabel 1 |
| Results of syntheses | 20a | For each synthesis, briefly summarise the characteristics and risk of bias among contributing studies. | Results |
|  | 20b | Present results of all statistical syntheses conducted. If meta-analysis was done, present for each the summary estimate and its precision (e.g. confidence/credible interval) and measures of statistical heterogeneity. If comparing groups, describe the direction of the effect. | Results |
|  | 20c | Present results of all investigations of possible causes of heterogeneity among study results. | Results |
|  | 20d | Present results of all sensitivity analyses conducted to assess the robustness of the synthesized results. | Table 1 |
| Reporting biases | 21 | Present assessments of risk of bias due to missing results (arising from reporting biases) for each synthesis assessed. | Results |

| **Section and Topic** | **Item #** | **Checklist item** | **Location**  **where item is reported** |
| --- | --- | --- | --- |
| Certainty of evidence | 22 | Present assessments of certainty (or confidence) in the body of evidence for each outcome assessed. | Results |
| **DISCUSSION** | | |  |
| Discussion | 23a | Provide a general interpretation of the results in the context of other evidence. | Discussion |
|  | 23b | Discuss any limitations of the evidence included in the review. | Discussion |
|  | 23c | Discuss any limitations of the review processes used. | Discussion |
|  | 23d | Discuss implications of the results for practice, policy, and future research. | Discussion |
| **OTHER INFORMATION** | | |  |
| Registration and protocol | 24a | Provide registration information for the review, including register name and registration number, or state that the review was not registered. | Method |
|  | 24b | Indicate where the review protocol can be accessed, or state that a protocol was not prepared. | Method |
|  | 24c | Describe and explain any amendments to information provided at registration or in the protocol. | - |
| Support | 25 | Describe sources of financial or non-financial support for the review, and the role of the funders or sponsors in the review. | Funding |
| Competing interests | 26 | Declare any competing interests of review authors. | Competing interests |
| Availability of data, code and other materials | 27 | Report which of the following are publicly available and where they can be found: template data collection forms; data extracted from included studies; data used for all analyses; analytic code; any other materials used in the review. | Method |

# Supplement Table 2. MOOSE Checklist

| **Item No** | **Recommendation** | **Reported on Page No** |
| --- | --- | --- |
| Reporting of background should include | | |
| 1 | Problem definition | 3-5 |
| 2 | Hypothesis statement | - |
| 3 | Description of study outcome(s) | Table 1 |
| 4 | Type of exposure or intervention used | 6-8 |
| 5 | Type of study designs used | PROSPERO |
| 6 | Study population | 6-7 |
| Reporting of search strategy should include | | |
| 7 | Qualifications of searchers (eg, librarians and investigators) | Title page |
| 8 | Search strategy, including time period included in the synthesis and key words | 6-7 |
| 9 | Effort to include all available studies, including contact with authors | 8-9 |
| 10 | Databases and registries searched | 8 |
| 11 | Search software used, name and version, including special features used (eg, explosion) | 10 |
| 12 | Use of hand searching (eg, reference lists of obtained articles) | Table 1 |
| 13 | List of citations located and those excluded, including justification | 8 |
| 14 | Method of addressing articles published in languages other than English | 8 |
| 15 | Method of handling abstracts and unpublished studies | 8 |
| 16 | Description of any contact with authors | - |
| Reporting of methods should include | | |
| 17 | Description of relevance or appropriateness of studies assembled for assessing the hypothesis to be tested | 7 |
| 18 | Rationale for the selection and coding of data (eg, sound clinical principles or convenience) | 7 |
| 19 | Documentation of how data were classified and coded (eg, multiple raters,  blinding and interrater reliability) | 7 |
| 20 | Assessment of confounding (eg, comparability of cases and controls in studies where appropriate) | 7-8 |
| 21 | Assessment of study quality, including blinding of quality assessors,  stratification or regression on possible predictors of study results | 7-8 |
| 22 | Assessment of heterogeneity | 8 |
| 23 | Description of statistical methods (eg, complete description of fixed or random effects models, justification of whether the chosen models account for predictors of study results, dose-response models, or cumulative meta- analysis) in sufficient detail to be replicated | 7-8 |
| 24 | Provision of appropriate tables and graphics | Figure1 Table 1 |
| Reporting of results should include | | |

| 25 | Graphic summarizing individual study estimates and overall estimate | Figs 2-3 |
| --- | --- | --- |
| 26 | Table giving descriptive information for each study included | Suppl eTable 11 |
| 27 | Results of sensitivity testing (eg, subgroup analysis) | Suppl  eTable 14 |
| 28 | Indication of statistical uncertainty of findings | 12-13 |
| Reporting of discussion should include | | |
| 29 | Quantitative assessment bias | Table 2,13,16 |
| 30 | Justification for exclusion | 8 |
| 31 | Assessment of quality of included studies | 8,Table 1 |
| Reporting of conclusions should include | | |
| 32 | Consideration of alternative explanations for observed results | 13-16 |
| 33 | Generalization of the conclusions | 17 |
| 34 | Guidelines for future research | 17 |
| 35 | Disclosure of funding source | 18 |

#

# Supplement Table 3. Risk of bias (quality) assessment using modified Newcastle-Ottawa Scale for cross-sectional and cohort studies

| **Newcastle-Ottawa Scale Criteria** | **Maximum Score** |
| --- | --- |
| *Cross-Sectional Studies* | |
| Sample representative of target sample (e.g. all eligible or random sample)? | 2 |
| Sample size justified and satisfactory? | 1 |
| Non-response rate is defined satisfactory. and characteristics of responders/non-responders compared? | 1 |
| Ascertainment of exposure (i.e. menstrual cycle) is valid and/or well described? | 1 |
| Assessment of outcome with robust tool and/or record linkage? | 2 |
| Outcome per group reported appropriately? | 1 |
| *Cohort Studies* | |
| Representativeness of exposed cohort (e.g. total population or random sample. selected group) | 1 |
| Method used to ascertain exposure (menstrual cycle phase) is robust? | 1 |
| Exposed and unexposed are matched or adjustment for confounding factors? | 2 |
| Assessment of outcome was blind to exposure status or used record linkage. were robust tools used? | 2 |
| Follow-up period was sufficiently long for outcomes to occur (e.g. more than one menstrual cycle? | 1 |
| Loss to follow-up rate is reported. low (<30%). and same in exposed and non-exposed? | 1 |

# Supplement Table 4. List of all tests and outcome measures used

| **Neurocognitive domain** | **Test and outcome measure** |
| --- | --- |
| **Processing speed** | Trail Making Test – A  Digit symbol coding  Stroop color word reading (Stroop W)  Stroop color naming task (Stroop C)  Wechsler Adult Intelligence Scale – Coding  CANTAB-Rapid Visual Information Processing |
| **Verbal learning** | Hopkins Verbal Learning Test-Revised  California Verbal Learning Test I/II (CVLT)  Controlled Oral Word Association [COWA] test  Cambridge Neuropsychological Test Automated Battery - Paired Associates Learning test  Wechsler Memory Scale - Logical memory, category verbal fluency 、Matrix Reasoning  The Rey Auditory Verbal Learning Test  COWAT- phonemic and semantic word finding  Wechsler Adult Intelligence Scale–Third Edition Vocabulary Matrix Reasoning  letter fluency semantic fluency  Vocabulary |
| **Visual learning** | Wechsler Intelligence Scale for Children—Third Edition- Visual reproduction, Faces recognition  Wechsler Adult Memory Scale - Visual reproduction  Block Design |
| **Working memory** | Cambridge Neuropsychological Test Automated Battery - Spatial Working Memory  Brief Assessment of Cognition in Schizophrenia Symbol - Digit Sequence  Wechsler Memory Scale - Spatial Span – 3rd ed.  Cambridge Neuropsychological Test Automated Battery - Spatial Working Memory\Sternberg working memory  Letter Number Span  N-back, 2-back  Wechsler Adult Intelligence Scale - Digit Span   - AX-CPT long delay - Paced Auditory Serial Addition Test - N-back, 1-back - Sternberg Working Memory task - Digit span test |
| **Attention** | - Continuous Performance Test - Identical Pairs (CPT- IP) - Continuous Performance Test: d´ - Continuous Performance Test: A   Continuous Performance Test Hearing   - Continuous Performance Test VISION   Monotone counting test |
| **Executive function** | - Trail Making Test – B   Cambridge Neuropsychological Test Automated Battery - Stockings of Cambridge  CANTAB IED  CANTAB SOC  CANTAB Information Sampling Task(  COWA Raw Score   - Cambridge Neuropsychological Test Automated Battery - Intradimensional/Extradimensional set shifting - Cambridge Neuropsychological Test Automated Battery - Stockings of Cambridge - Wisconsin Card Sorting Test - Wisconsin Card Sorting Test perseverative errors - Wisconsin Card Sorting Test percentage perseverative errors - Wisconsin Card Sorting Test total errors |

Supplement Table 5. Verbal learning subgroup meta-analyses

| \| Subgroup \| \| --- \| | Tests included | \| k \| \| --- \| | Pooled Hedges’ g (95% CI) | \| *p* \| \| --- \| | Meta-analyzed |
| --- | --- | --- | --- | --- | --- | --- | --- | --- |
| List-learning — Immediate | HVLT/CVLT/RAVLT (Immediate or Total learning) | 6 | −0.558 (−1.099 to −0.017) | **0.043** | Yes |
| List-learning — Delayed | HVLT/CVLT/RAVLT (Delayed recall) | 3 | −0.296 (−0.526 to −0.067) | **0.011** | Yes |
| Logical Memory — Immediate (WMS LM-I) | WMS Logical Memory I (Immediate) | <3 | - | - | NO |
| Logical Memory — Delayed (WMS LM-II) | WMS Logical Memory II (Delayed) | <3 | - | - | NO |

**Footnotes.** Effects are Hedges’ g coded as **CHR − FHR** (negative = poorer CHR; favours FHR). Random-effects model (DerSimonian–Laird). One effect per study; where multiple outcomes occurred within a study/domain, a within-study fixed-effects composite (**r = 0.50**) was used. Subgroups with **k < 3** were not meta-analysed; single-study effects are described narratively in the Supplement.

**Abbreviations.** HVLT = Hopkins Verbal Learning Test–Revised; CVLT = California Verbal Learning Test; RAVLT = Rey Auditory Verbal Learning Test; WMS = Wechsler Memory Scale; LM-I = Logical Memory Immediate; LM-II = Logical Memory Delayed; CHR = Clinical High Risk; FHR = Familial High Risk.


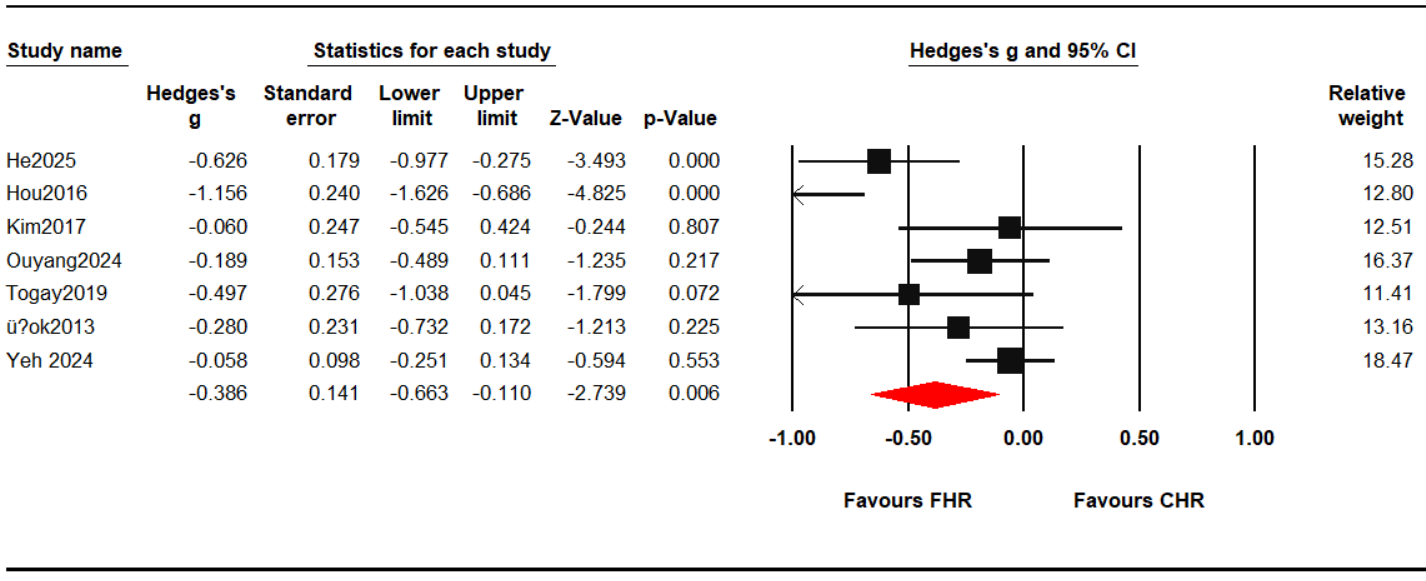


Supplement Figure1. TMT-A CHR vs FHR. Random‐effects meta-analysis of Hedges’ g (CHR−FHR); negative values favor FHR. The diamond shows the pooled effect with 95% CI. Prediction interval (95%): [-1.281, 0.501].


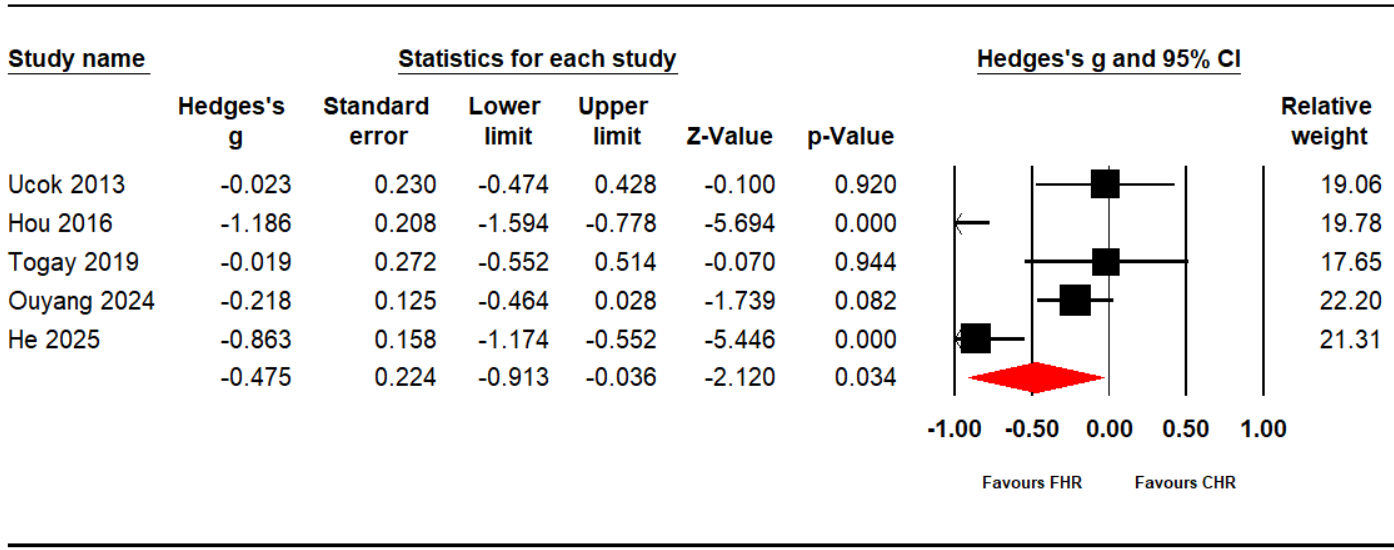


Supplement Figure 2. SWCT CHR vs FHR. Random‐effects meta-analysis of Hedges’ g (CHR−FHR); negative values favor FHR. The diamond shows the pooled effect with 95% CI. Prediction interval (95%): [-2.098, 1.149].


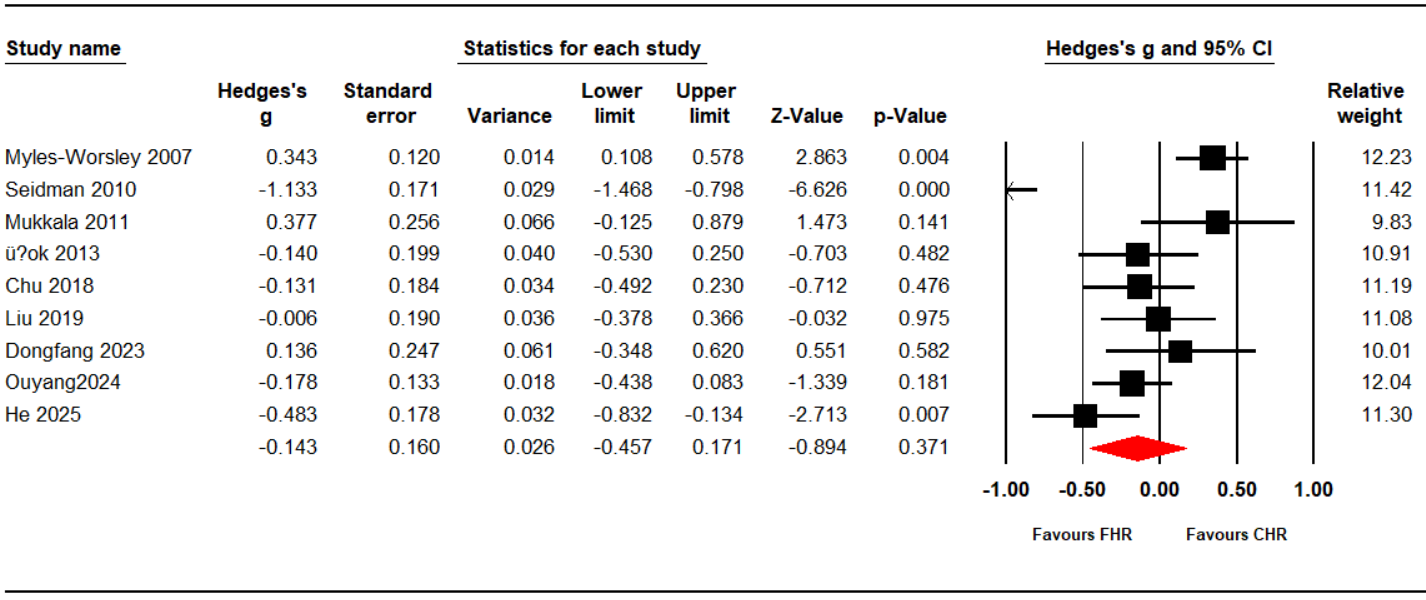


Supplement Figure 3. Attention CHR vs FHR. Random‐effects meta-analysis of Hedges’ g (CHR−FHR); negative values favor FHR. The diamond shows the pooled effect with 95% CI. Prediction interval (95%): [-1.256, 0.969].


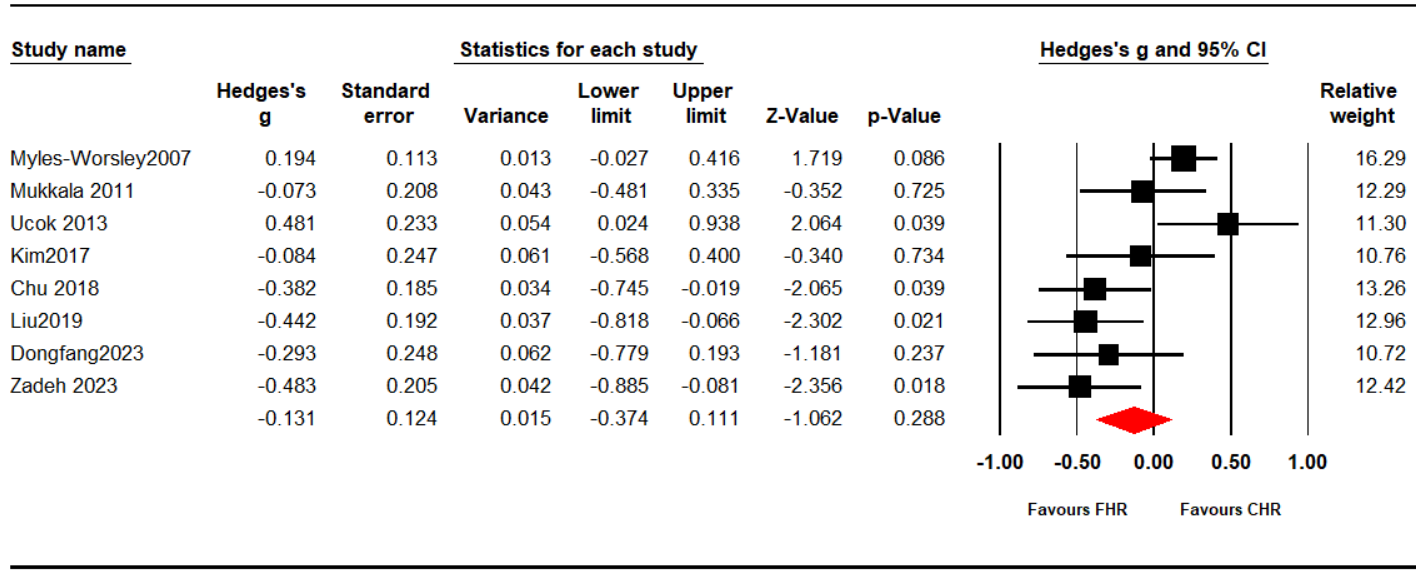


Supplement Figure 4. Working memory CHR vs FHR. Random‐effects meta-analysis of Hedges’ g (CHR−FHR); negative values favor FHR. The diamond shows the pooled effect with 95% CI. Prediction interval (95%): [-0.892, 0.629].


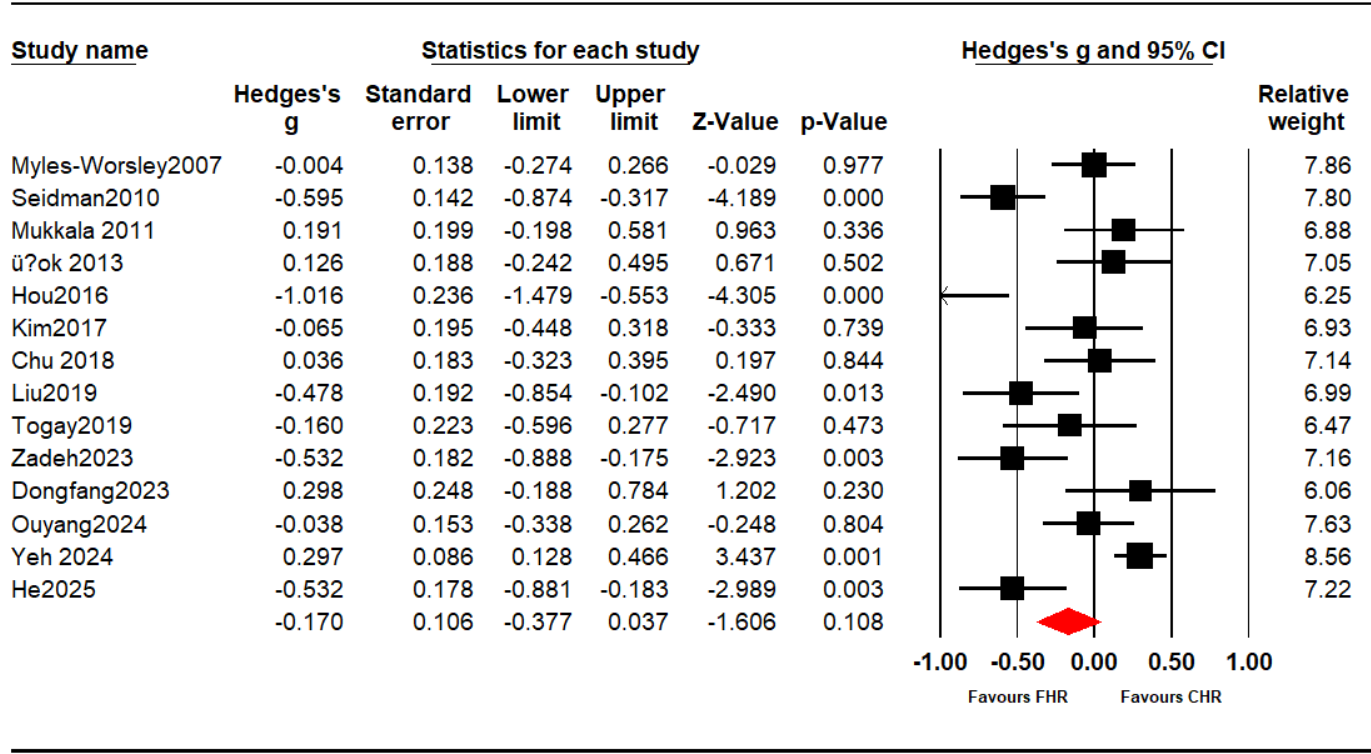


Supplement Figure 5. Executive function CHR vs FHR. Random‐effects meta-analysis of Hedges’ g (CHR−FHR); negative values favor FHR. The diamond shows the pooled effect with 95% CI. Prediction interval (95%): [-0.968, 0.628].


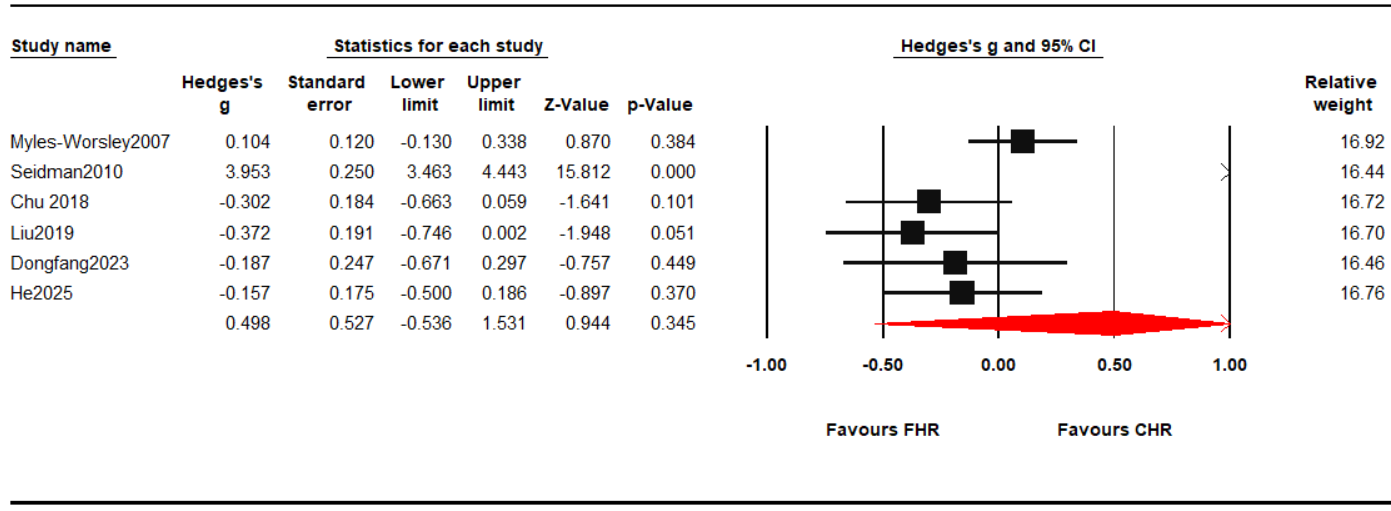


Supplement Figure 6. Visiable learning CHR vs FHR. Random‐effects meta-analysis of Hedges’ g (CHR−FHR); negative values favor FHR. The diamond shows the pooled effect with 95% CI. Prediction interval (95%): [-3.336, 4.332].


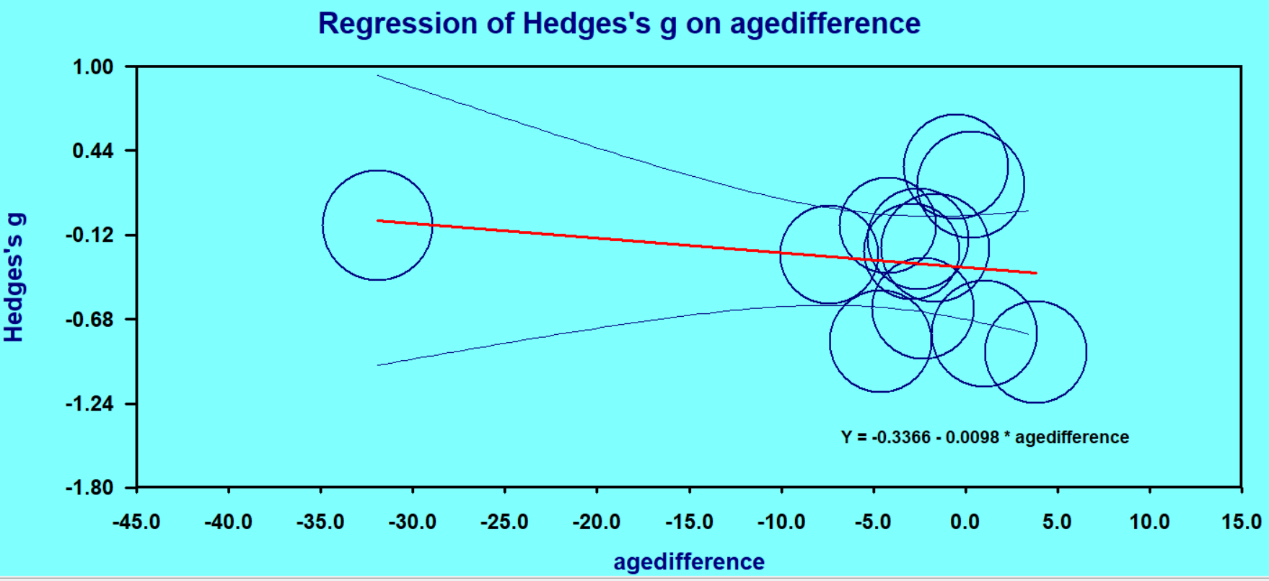


Supplement Figure 7**. Meta-regression for processing speed by age.**


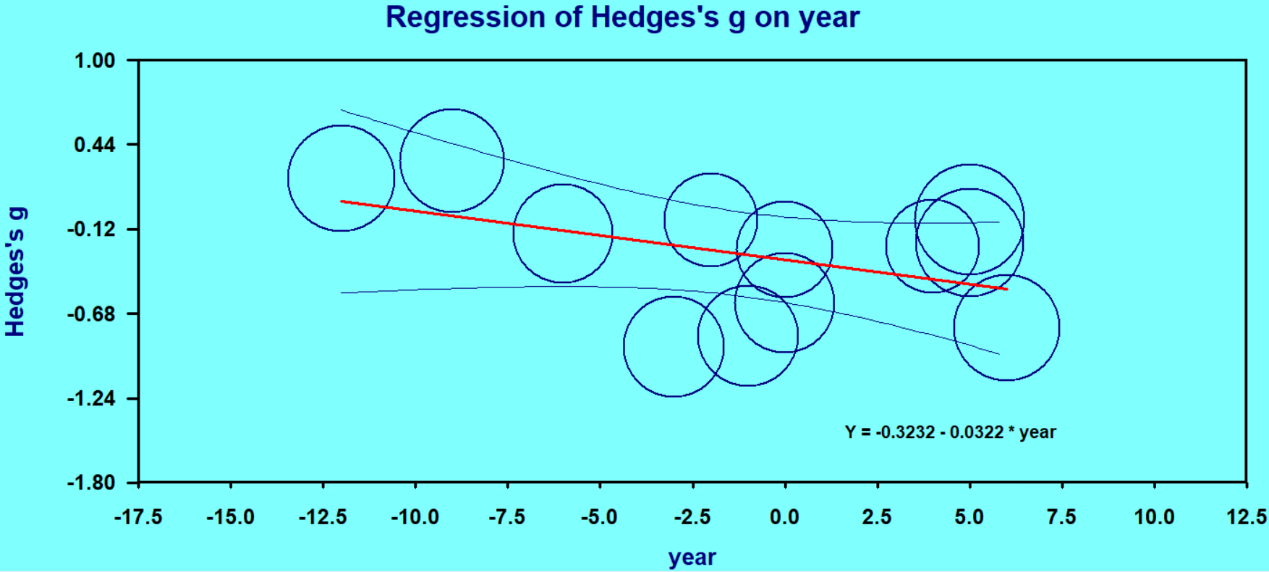


Supplement Figure 8**. Meta-regression for processing speed by year centred**


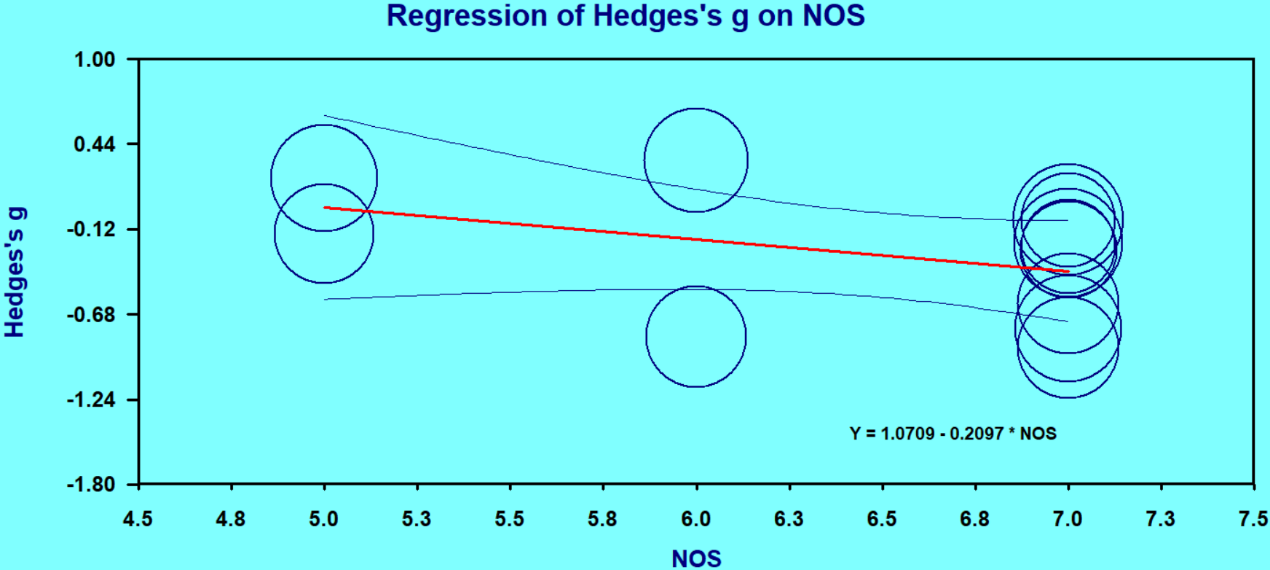


Supplement Figure9. Meta-regression for processing speed by NOS
